# Perspective: Beta Cell Glucose Toxicity in Type 2 Diabetes: Nrf2 and the Endogenous Antioxidant Response

**DOI:** 10.3390/antiox15080997

**Published:** 2026-08-12

**Authors:** Roderick Paul Robertson

**Affiliations:** Division of Metabolism, Endocrinology, and Nutrition, Department of Internal Medicine, The University of Washington, Seattle, WA 98195, USA; rpr@uw.edu

**Keywords:** beta cell, glucose toxicity, type 2 diabetes, Nrf2, antioxidant response

## Abstract

Increasing attention is being given to the concept that mild hyperglycemia in patients with early type 2 diabetes initiates a vicious cycle in which progressively higher levels of glycemia generate higher levels of reactive oxygen species (ROS) both in the general blood circulation and within the beta cells themselves. Theoretically, over time, this might worsen already damaged beta cell function (glucose toxicity) and augment progression to frank hyperglycemia. At issue is whether in the early stages of type 2 diabetes the beta cell is simply a passive victim in this scenario or whether it might try to mount an endogenous antioxidant response to defend itself against local excessive ROS formation. Since Nrf2 is the primary regulator of endogenous antioxidant synthesis, a greater understanding of Nrf2 activity in beta cells undergoing hyperglycemic stress may lead to new therapeutic insights for type 2 diabetes.

## 1. Diabetes and Oxidative Stress

Early clinical studies pointed out associations between oxidative stress and chronic complications of type 2 diabetes [1,2,3,4]. One possible mechanism of this is that chronically high concentrations of glucose have adverse effects on insulin gene expression. That this might be the case was suggested by experiments with cultures of the beta cell line HIT-T15 in which chronic exposure to high glucose levels, but not low glucose levels, decreased insulin gene transcription and altered binding of a gene regulatory protein termed STF-1, now termed PDX-1. Subsequent studies using both HIT-T15 cells and BTC-6 beta cell lines demonstrated that inactivation of another insulin gene regulatory factor, then termed RIPE3b1 activator, now termed MAFA, might also be involved [5].

**Figure 1:**
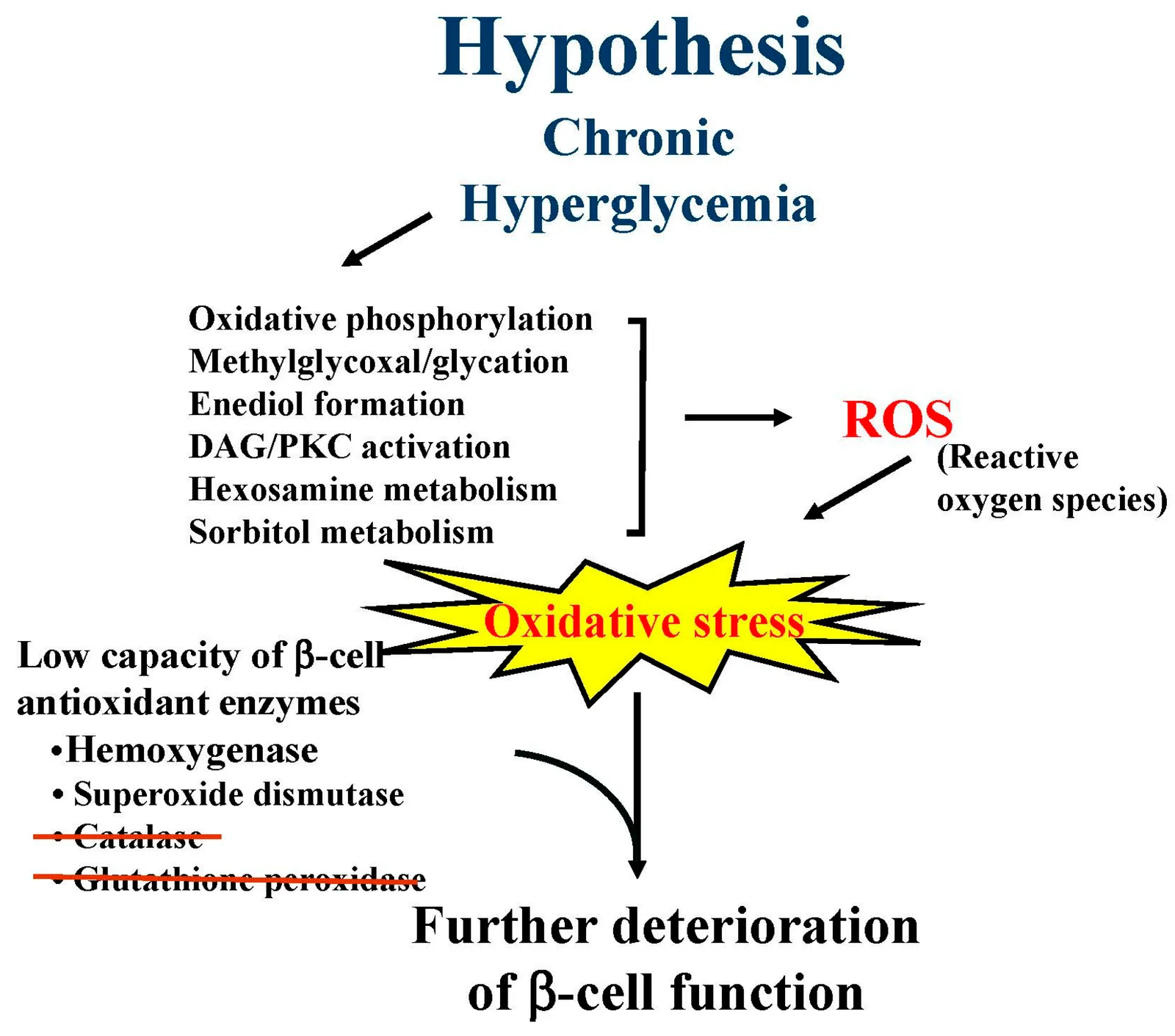
Hypothesis wherein initial mild elevations of blood glucose associated with subjects destined to develop type 2 diabetes eventually lead to constant hyperglycemia and toxic levels of reactive oxygen species (ROS) via various metabolic pathways. This in turn leads to greater damage to beta cells, further failure of beta cell function, and frank hyperglycemia. Beta cells inherently have limited protection against ROS by virtue of the absence of intracellular glutathione peroxidase and catalase, which facilitates this progression of glucose-induced damage to themselves. The red lines crossing out the words catalase and glutathione peroxidase, which are normally present in other cells, are meant to indicate their absence in beta cells.

Oxidative stress due to chronic hyperglycemia is a likely mechanism for deleterious effects on insulin gene expression because metabolism of glucose uses pathways that generate reactive oxygen species (ROS). The pathways include glucose autooxidation, protein kinase C activation, methylglyoxal formation and glycation, hexosamine metabolism, sorbitol formation, and oxidative phosphorylation, all of which can generate increased levels of ROS (Figure 1) [5]. Using this information as a rationale, Tanaka et al. [6] examined whether the antioxidants n-acetylcysteine or aminoguanidine protect diabetes-prone Zucker diabetic rats (ZDF) fed a high-fat diet from developing diabetes over a six-week period. Both drugs prevented a rise in blood oxidative stress markers (8-hydroxy-2′-deoxyguanosine and malondialdehyde + 4-hydoxy-2-noneal) and partially prevented hyperglycemia, glucose intolerance, and defective insulin secretion as well as decrements in beta cell insulin content, insulin gene expression, and PDX-1 binding to the insulin gene promoter in the animals.

When considering strategies to protect pancreatic beta cells from oxidative stress, an important factor is that, uniquely, beta cells do not have the entire array of endogenous antioxidants seen in most mammalian tissues. Specifically, it has been reported by several groups that beta cells have only low to absent catalase and glutathione peroxidase (GPx) levels, although superoxide dismutase and hemoxygenase appear to be amply present (Figure 1) [7,8,9]. This unique circumstance causes beta cells to be especially susceptible to ROS and oxidative stress. In this regard, Tanaka et al. observed that high glucose concentrations increased intracellular peroxide levels in human islets [10] and that inhibition of gamma-glutamyl cysteine synthetase by buthionine sulfoximine augmented an increase in islet peroxide induced by ribose and a decrease in insulin mRNA levels, content, and secretion in both islets and the cultured HIT-T15 beta cell line. Moreover, adenoviral overexpression of GPx protected beta cells from the adverse effects of ribose.

## 2. Nrf2 and Regulation of Antioxidants in Beta Cells

**Figure 2:**
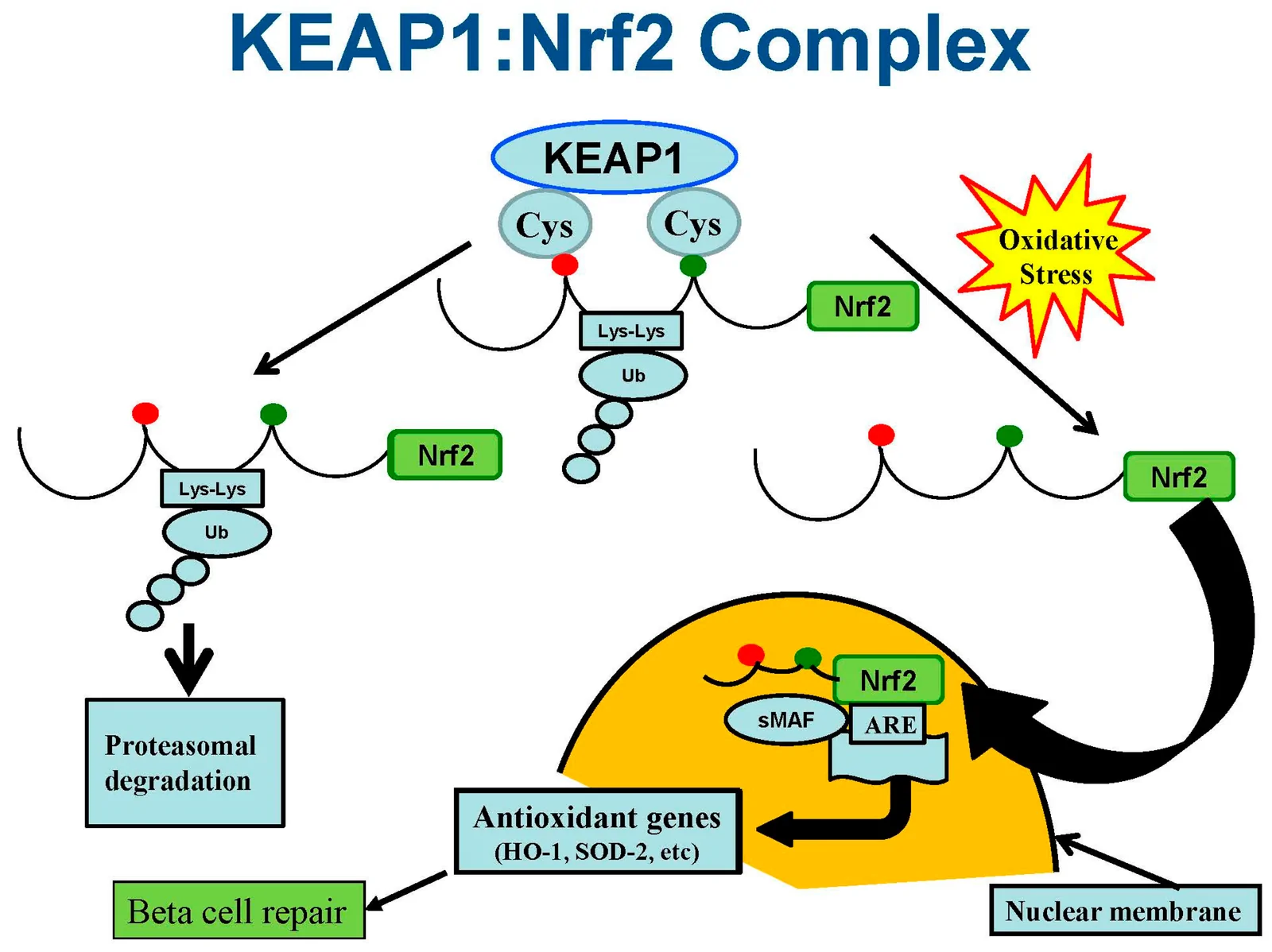
Illustration of the Keap1:Nrf2 complex with two metabolic routes. In the quiescent state the complex is routed to the proteasome for degradation. Under conditions of oxidative stress, Nrf2 dissociates from KEAP1 and translocates into the nucleus where, in the presence of sMAF, it stimulates the synthesis of antioxidant enzymes, in this case HO-1.

Nrf2, or nuclear factor erythroid 2-related factor, is generally considered to be the master regulator of endogenous antioxidant synthesis and plays a central role in regulating the synthesis of a wide variety of antioxidant genes in mammals. Nrf2 resides in the cytosolic compartment and in its inactive state is bound to Keap1 (Kelch-like ECH-associated protein; Figure 2), which plays a key role in regulating Nrf2 activity [11]. Keap1 exists as a dimer inside beta cells and interacts with the Cul3/Rbx1-based E3-ubiquitin ligase complex with Nrf2, leading to its continuous ubiquitination and proteasomal degradation. When cells encounter stress from oxidants, Nrf2 dissociates from Keap1 and translocates to the nucleus to bind to the antioxidant response element, which leads to increased transcription of antioxidant genes. Kumar et al. reported that induction of Nrf2 was required for ChREBP alpha-mediated mitochondrial biogenesis and for glucose-stimulated and ChREB-alpha-augmented beta cell proliferation [12]. Nrf2 also modulates inflammatory responses by inhibiting pro-inflammatory genes and mediates cell death pathways, including apoptosis and ferroptosis, as described in the review by Yang et al. [13]. This review is highly recommended for readers interested in an exhaustive review of Nrf2 biochemistry and biology, as well as information about targeting epigenic and post-translational modifications of Nrf2 as potential therapeutic avenues for treating various diseases.

Shalev has emphasized that Nrf2 also regulates thioredoxin pathways [14] in addition to the glutathione pathway. Mammalian peroxiredoxins (Prdx1-6) are ubiquitously expressed in all cells including pancreatic beta cells. Prdx1-6 detoxifies H_2_O_2_ and reduces organic hydroperoxides, primarily by oxidation of their cysteine residues. Diabetes, glucose, and cellular stresses induce thioredoxin-interacting protein (Txnip) expression, which can interact with thioredoxins to inhibit their antioxidative and antiapoptotic effects. TXNIP has been suggested to be an important link between glucose toxicity and beta cell death by Chen et al. [15].

## 3. Relevance of Nrf2 Pharmacology to Type 2 Diabetes and Beta Cell Biology

**Figure 3:**
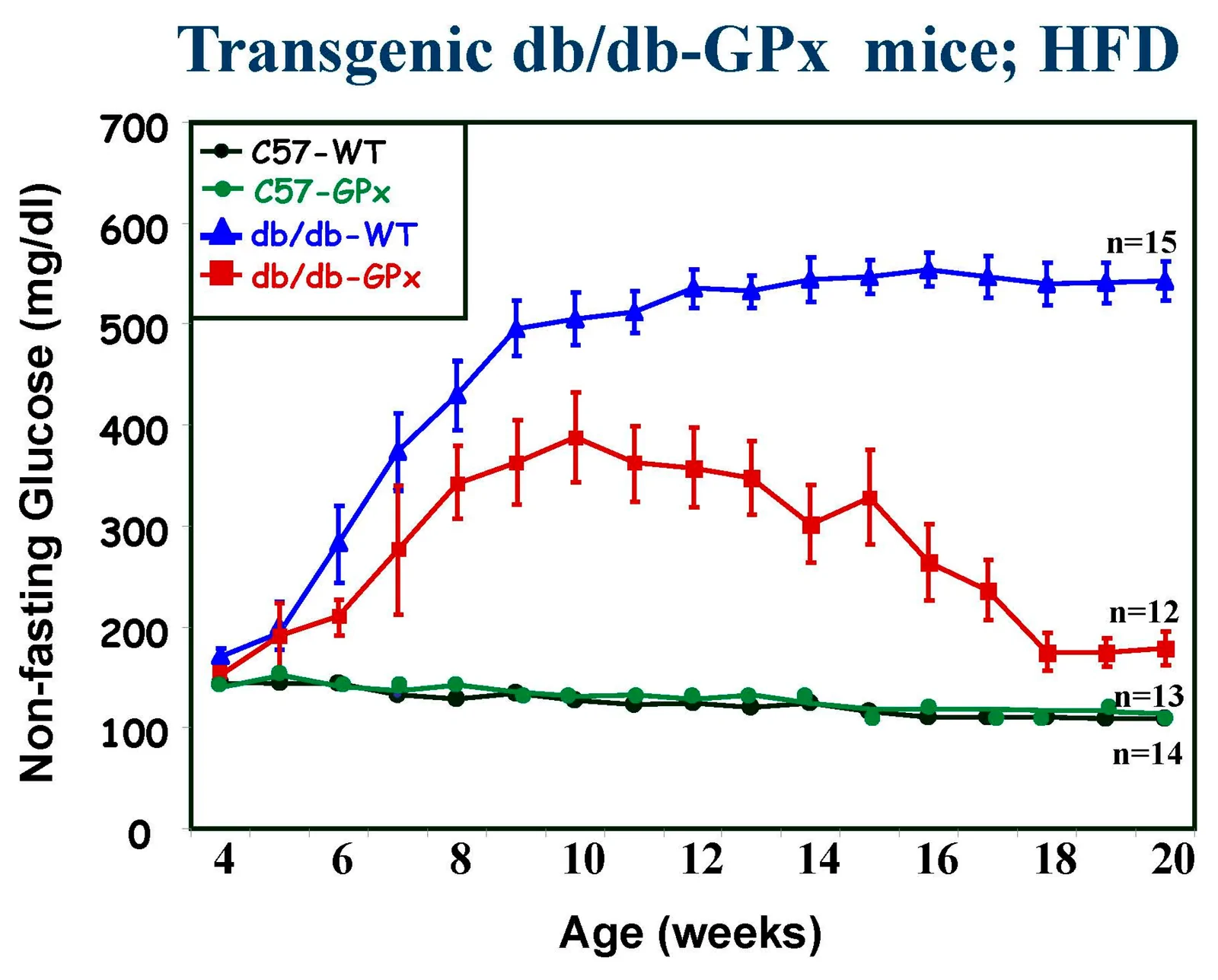
Comparison of non-fasting blood glucose levels in four groups of mice fed a high-fat diet. (1) Wild-type C57 mice; (2) wild-type C57 mice transgenic for glutathione peroxidase (GPx); (3) wild-type db/db mice with GPx deficiency; and (4) wild-type db/db mice transgenic for GPx. Wild-type C57 mice and the wild-type C57 mice transgenic for GPx fed HFD did not develop hyperglycemia. Wild-type db/db mice fed HFD developed sustained hyperglycemia. Wild-type db/db-GPx mice initially became moderately hyperglycemic, but by 8 weeks, their glucose levels plateaued and then became normal (taken from Ref. [16]).

Knowledge that beta cells do not have appreciable levels of glutathione peroxidase led to studies of the effects of beta cell overexpression of GPx in vivo in diabetes-prone animals who reliably become hyperglycemic when fed a high-fat diet (HFD). Harmon et al. (Figure 3 [16]) used HFD-sensitive db/db mice to determine whether transgenic increases in the levels of endogenous GPx-1 in beta cells specifically could protect against the hyperglycemic effects of the HFD. In these studies, glucose levels in the control mice rose and remained elevated throughout the 20-week study. Glucose levels in the db/db-GPx(+) mice also initially rose modestly but then at 10 weeks fell back into a normal range (Figure 2). This suggested a complex scenario in which the HFD initially caused beta cell damage and high blood glucose levels, but also the consequent higher glucose levels stimulated the human insulin promoter of the GPx transgene that increased GPx activity, which in turn repaired beta cells and increased insulin secretion, and thereby lowered glucose levels.

**Figure 4:**
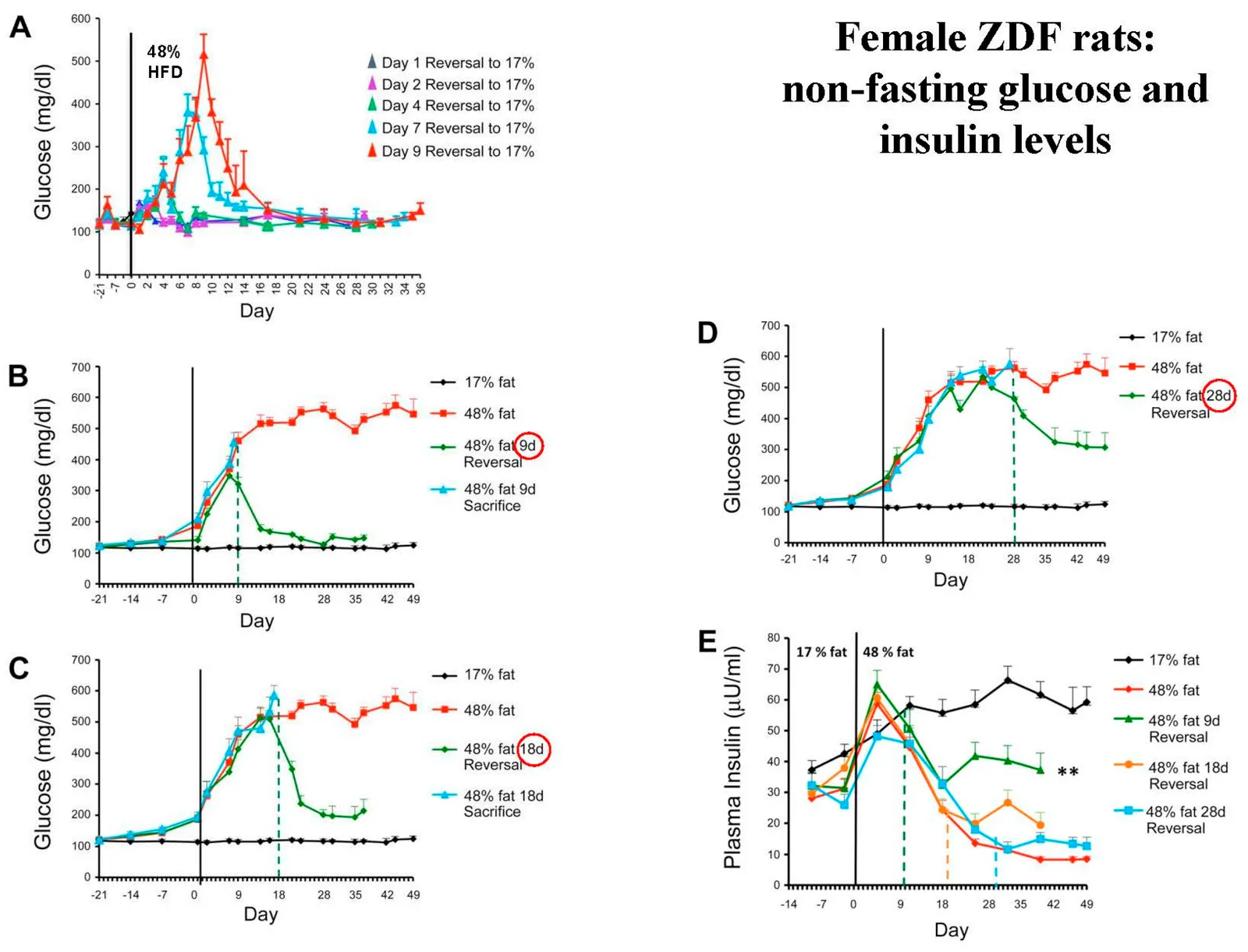
Comparison of non-fasting blood glucose and insulin levels in female ZDF rats during induction of hyperglycemia by feeding with HFD followed by switching to regular diet after increasing durations of feeding with the HFD. The exposure to HFD were 1, 2, 4, 7, 9, 18, and 28 days prior to switching back to regular diet for two weeks. The degrees of hyperglycemia were progressively worse and the returns toward normoglycemia were progressively slower as the length of exposure to the high-fat diets increased (taken from Ref. [17]). Panel (**A**). Plots of daily glucose levels before and after feeding HFD for the indicated duration times before discontinuation of HFD and returning to regular diets. Panel (**B**). Plots of daily glucose levels before and after feeding HFD to two subgroups, one of which was sacrificed at 9 days or allowed to live for 48 days. Panel (**C**). Plots of daily glucose levels before and after feeding HFD to two subgroups, one of which was sacrificed at 18 days or allowed to live for 48 days. Panel (**D**). Plots of daily glucose levels before and after feeding HFD to two subgroups, one of which was sacrificed at 28 days or allowed to live for 48 days. Panel (**E**). Plots of daily insulin levels in the respective groups in panels (**B**–**D**) that were or were not sacrificed on days 9, 18, or 28 days. ** *p* < 0.01.

**Figure 5:**
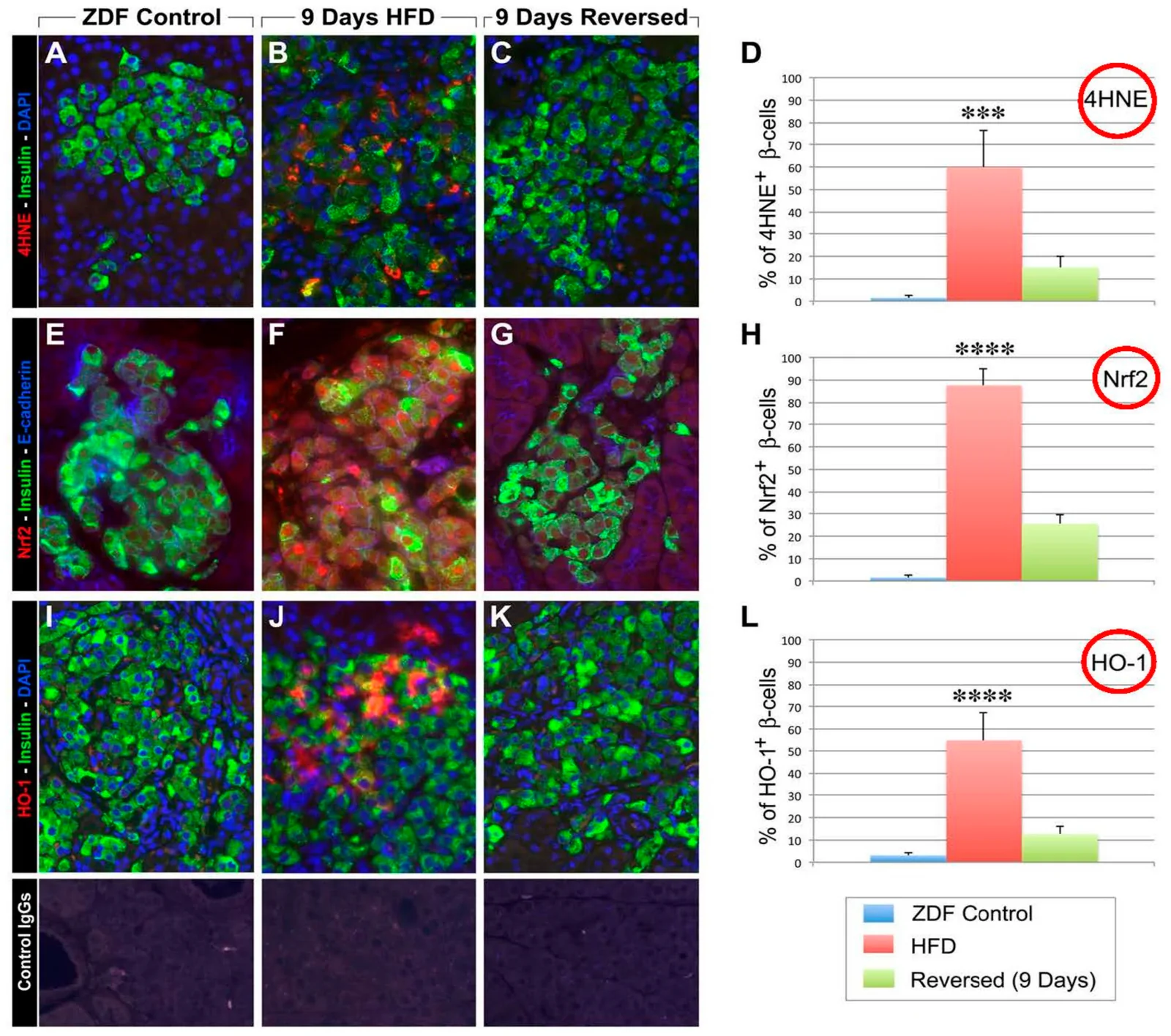
Pancreatic sections of ZDF rats described in Figure 3 were double-stained for 4-HNE (a marker for oxidative stress), Nrf2 (the gene that regulates synthesis of antioxidants), and the antioxidant hemoxygnase-1 (**A**–**C**,**E**–**G**,**I**–**K**). Compared to control values, Nrf2 (**H**), hemoxygenase-1 (**L**), and 4HNE levels (**D**) increased by 9 days of feeding on a high-fat diet. In rats that were fed a high-fat diet but returned to their normal diet for 2 weeks, levels of 4HNE, Nrf2, and HO-1 returned to control levels. Statistical differences in panels shown in (**D**,**H**), and L are *** *p* < 0.001 and *p* < 0.0001 **** (taken from Ref. [17]).

These findings raised the question of whether beta cells undergo oxidative stress over time in an attempt to self-repair via localized antioxidant production, and if so, how soon should exogenous treatment with antioxidants be started in the course of the disease to provide help in this effort? Answers to these questions could provide valuable information about how soon in type 2 diabetes antioxidant-based treatment needs to be started. Abebe et al. [17] conducted studies with female Zucker diabetic fatty rats fed an HFD for 1, 2, 4, 7, 9, or 28 days, followed by a return to regular chow for 2 weeks (Figure 4). Beta cell repair was defined as reversal of elevated glucose levels and low blood insulin levels caused by the HFD, as well as reversal of beta cell microscopic structural damage visualized by imaging studies. Evidence of functional beta cell damage was observed after 9 days of HFD followed by repair after 2 weeks of return to normal chow (Figure 4). However, after a longer duration of 18 and 28 days, as opposed tothe 9-day exposure to HFD, damage was more severe and repair was less evident. Beta cell mass was 4-fold greater after 9 days but only 3-fold greater after 18 days, with no increase after 28 days of HFD followed by 2 weeks of normal chow. No changes were observed in beta cell apoptosis or replication in any of the animals. HFD was associated with formation of intracellular markers of oxidative stress, intracellular translocation of Nrf2, and induction of hemoxygenase via the Nrf2/oxidant pathway (Figure 5). Flow cytometry-based assessment of beta cell volume, morphology, and insulin-specific immunoreactivity as well as structural analysis by transmission electron microscopy revealed that short-term exposure to HFD produced significant changes in beta cell morphology and function that were reversible after returning to regular chow. However, Nrf2 activation of self-repair was not evident with longer exposure to hyperglycemia. These findings pointed to activation of the Nfr2/antioxidant pathway as an early mechanism mediating the ability of beta cells to self-repair after a short but not extensive exposure to HFD. This time-related feature suggests that earlier rather than later intervention for hyperglycemia in the development of full blown T2D is the better therapeutic choice.

The information that demonstrated that genetic provision of GPx in vitro to beta cells and in vitro to animals ameliorated the development of beta cell damage [10,11] led to the work by Mahadevan et al. [18], who reported the results from the treatment of diabetic rats with the oral antioxidant Ebselen, a glutathione peroxidase mimetic. Male ZDF (ZDF-Leprfa/fa) glucose-sensitive rats were treated or not treated at 6 weeks of age with oral Ebselen gavage and fed HFD. Ebselen prevented islet apoptosis, maintained beta cell Pdx-1 and MafA levels, and preserved beta cell mass and function. Since Ebselen is an oral therapeutic antioxidant previously used in human clinical trials [19,20,21,22,23,24], it is a novel therapeutic candidate for ameliorating fasting hyperglycemia and further deterioration of beta cell mass and function in humans at the onset of type 2 diabetes. In this context, it is important to point out that several years after the manuscript by Mahadevan et al. [18] was published, Beckman et al. reported that Ebselen did not improve oxidative stress or glycemia in 10 subjects with type 2 diabetes [25]. However, the dose of Ebselen used and/or drug compliance appeared to be suboptimal since it also did not affect the subjects’ oxidative stress profile nor endothelium-dependent vasodilation.

## 4. Clinical Management of Type 2 Diabetes

At the clinical level, the management of Type 2 diabetes often involves a period of “wait and see” over months to determine if hyperglycemia spontaneously resolves. Our data [17] support the concept that early rather than delayed treatment of hyperglycemia in new onset type 2 diabetes in humans would be the better course for treatment of remaining beta cells. However, trials using various oral antioxidants to treat existing hyperglycemia in humans with type 2 diabetes have been largely unsuccessful. Why is this? On one hand, Dodson et al. [25] in a review pointed out reports [26,27,28] that administration of the Nrf2 inducer cinnamaldehyde attenuated nephropathy in Nrf2+/+ mice injected with streptozotocin and that oral administration of naringenin to streptozotocin-injected mice reduced pancreatic ROS production and beta cell apoptosis, restoring insulin production and glucose tolerance, in part through increased activity of superoxide dismutase catalase and GPx. On the other hand, a problem with using classical oral antioxidants is that they act as hydrogen-donating or electron-donating agents designed to quench ROS. It is perhaps not surprising that scavenging of ROS by oral antioxidants would be ineffective because endogenous ROS react extremely rapidly to nearby biological targets and outcompete antioxidants taken orally. A more effective strategy might be the removal of ROS precursors, such as high glucose concentrations, through the early use of long-acting insulin, which is sometimes used in clinical practice (but not recommended by the American Diabetes Association). A major caveat regarding the line of reasoning that leads to the strategy of altering Nrf2-induced synthesis of antioxidants in beta cells was provided by Fu J et al. [29]. They pointed out that while it might maintain intracellular redox hemostasis and limit oxidative damage, manipulating Nrf2 pathways also has the potential to impede ROS that function as physiological signaling molecules and thereby diminish beta cell function.

In conclusion, this Perspective encourages clinical trials to fully evaluate whether antioxidant drugs can be used early in type 2 diabetes to protect beta cells from deterioration caused by continual worsening of hyperglycemia and constant endogenous production of ROS [30,31,32]. Since beta cells are deficient in GPx, one such drug is Ebselen, which is an oral GPx mimetic and has been shown in clinical trials to be safe and effective for other diseases [19,20,21,22,23,24]. It is not clear why controlled clinical trials with Ebselen for treatment in type 2 diabetes have not been embraced by the pharmaceutical industry.

## Data Availability

This manuscript is a scientific review and based solely on previously reported data with appropriate references.
